# De-problematizing masculinity among heterosexual African, Caribbean, and Black male youth and men

**DOI:** 10.17269/s41997-021-00596-3

**Published:** 2022-03-15

**Authors:** Josephine Etowa, Doris M. Kakuru, Akalewold Gebremeskel, Egbe B. Etowa, Bagnini Kohoun

**Affiliations:** 1 OHTN Chair in Black Women’s HIV Prevention and Care, School of Nursing, Faculty of Health Sciences, University of Ottawa, 451 Smyth Road, Ottawa, ON K1H 8M5 Canada; 2 School of Child and Youth Care, Faculty of Human and Social Development, University of Victoria, Victoria, BC Canada; 3 School of International Development and Global Studies, Faculty of Social Sciences, University of Ottawa, Ottawa, ON Canada; 4 Department of Sociology, Anthropology & Criminology, Faculty of Humanities and Social Sciences, University of Windsor, Windsor, ON Canada; 5 Canadians of African Descent Health Organization, Ottawa, ON Canada

**Keywords:** ACB men, Heterosexuality, HIV, Black men, Masculinity, Health, Ethnicity, Community, Hommes ACN, hétérosexualité, VIH, hommes noirs, masculinité, santé, appartenance ethnique, communauté

## Abstract

**Objectives:**

The dominant discourse in literature often constructs heterosexual African, Caribbean, and Black (ACB) masculinity as inherently problematic and in need of “correction, repair, or rescue.” This discourse privileges hegemonic male standards and conceals the power relations that shape racialized masculinities. Our study of self-identified heterosexual ACB men and male youth examines how performative and perceptual attenuations of hegemonic masculinity can moderate social and behavioural vulnerabilities in the context of HIV prevention, transmission, and survival.

**Methods:**

We used descriptive qualitative methods informed by community-based participatory research. Individual in-depth interviews and focus group discussions were conducted with 63 ACB men and male youth (aged 16 and above) residing in Ottawa, Canada, including community leaders, HIV service providers, and decision makers. The interviews were transcribed verbatim, and thematically analyzed with NVivo software. Member-checking, peer debriefing, and external audit ensured trustworthiness of data.

**Results:**

ACB men and male youth define masculinity by their ability to provide for, protect, love, and lead their families. Within ACB cultures, men demonstrate their masculinity through their traditional role as family breadwinners, and are expected to be strong, bold, and responsible. This positive view of masculinity is potentially beneficial to the well-being of ACB men and male youth, and challenges mainstream notions of Black masculinity as uncontrolled, risky, toxic, or even predatory.

**Conclusion:**

A positive view of masculinity among ACB heterosexual men and youth could support future practice and policy interventions aimed at strengthening community responses to HIV and health.

## Résumé

**Objectifs:**

Le discours dominant dans la littérature spécialisée construit souvent la masculinité hétérosexuelle africaine, caribéenne et noire (ACN) comme étant problématique en soi et nécessitant « correction, réparation ou sauvetage ». Ce discours privilégie les normes masculines hégémoniques et cache les relations de pouvoir qui façonnent des masculinités racisées. Notre étude auprès d’hommes ACN adultes et plus jeunes s’identifiant comme étant hétérosexuels cherche à déterminer si des atténuations performatives et perceptuelles de la masculinité hégémonique peuvent modérer les vulnérabilités sociales et comportementales dans le contexte de la prévention du VIH, de la transmission du virus et de la survie.

**Méthode:**

Nous avons utilisé des méthodes qualitatives descriptives éclairées par la recherche participative communautaire. Des entretiens individuels en profondeur et des groupes de discussion ont été menés avec 63 hommes ACN adultes et plus jeunes (16 ans et plus) vivant à Ottawa, au Canada, dont des responsables locaux, des dispensateurs de services en matière de VIH et des décideurs. Les entretiens ont été transcrits mot à mot, puis analysés par thème à l’aide du logiciel NVivo. Une vérification des membres, une séance-bilan avec des pairs et un audit externe ont assuré la fiabilité des données.

**Résultats:**

Les hommes ACN adultes et plus jeunes définissent la masculinité selon leur capacité de protéger, d’aimer et de diriger leur famille et de subvenir à ses besoins. Dans les cultures ACN, les hommes prouvent leur masculinité en jouant le rôle traditionnel de soutiens de famille et sont censés être forts, audacieux et responsables. Cette image positive de la masculinité, potentiellement favorable au bien-être des hommes ACN adultes et plus jeunes, remet en question la notion de la masculinité noire comme étant incontrôlable, risquée, toxique ou même prédatrice.

**Conclusion:**

Une image positive de la masculinité chez les hommes hétérosexuels ACN adultes et plus jeunes pourrait appuyer de futures interventions axées sur les pratiques et les politiques visant à renforcer la riposte communautaire au VIH et la santé.

## Introduction

Heterosexual African, Caribbean, and Black (ACB) men experience social inequalities that increase their vulnerabilities to HIV (Husbands et al., 2014; Husbands et al., 2020). Heterosexual ACB men and youth (young men) have largely been ignored in research and programming, as most HIV studies on ACB masculinity focus on men who have sex with men. Black people account for 2.5% of Canada’s population (PHAC, 2016) and less than 5% of the population of the province of Ontario (Remis et al., 2010), yet ACB people living with heterosexually contracted HIV account for 20% of HIV-positive cases in Ontario. In 2009, men were estimated to represent 60% (*n* = 3075) of the provincial total of ACB people living with HIV, a 40% (*n* = 885) increase from 2004 (Husbands et al., 2014). However, the 2018 national surveillance statistics (excluding Quebec) show a decline to 19.4% in terms of prevalence of heterosexually contracted HIV among males (PHAC, 2019), a trend reversal uncorrelated to Ontario’s HIV research, programming, and policy efforts, which remain inadequately aligned with the needs of heterosexual ACB men.

In this study, we recruited self-identified heterosexual ACB men and youth (aged 16 and over) with the help of trained peer recruiters through networks, venues, and events which they frequented, such as the AIDS Committee of Ottawa, River Jordan Ministries, Somerset West Community Health Centre (SWCHC), and Black Student Leaders Association, as well as the African, Caribbean, and Black (ACB) Community Multicultural Day event. Potential participants were invited to a series of consultative meetings to discuss Black men’s health. We were guided by the understanding that while support services are generally available to help people cope with HIV challenges, heterosexual men and youth may perceive their options as limited due to the negative discourse around them. When heterosexual males are held responsible for their female partners’ vulnerability to HIV, males may not access HIV-related services due to self-blame; the fear of being blamed, judged, or stigmatized; or the perception that their engagement in HIV services is under-valued (Antoniou et al., 2012). It is therefore important to recognize and accommodate the circumstances and priorities of ACB men (Bowleg & Raj, 2012), as well as to identify and highlight their potential resourcefulness.

The aim of our research is to generate knowledge to inform effective HIV responses which engage ACB communities, and to identify the individual and structural factors that promote resilience and reduce HIV-related vulnerabilities among heterosexual ACB men and youth. In the context of our inquiry, this essay reports on how Black heterosexual masculinity is understood, endorsed, resisted, or appropriated by ACB males, academics, and other stakeholders. The essay also critically interrogates negative views of ACB masculinity, as well as how our participants understand their vulnerability and resilience, especially in relation to HIV. Finally, we report on how participants generate self-affirming narratives that resist common gendered and sexually racialized interpretations of Black men’s heterosexuality and masculinity.

## Literature review

Previous studies on masculinity show that ACB men experience social inequalities that make them more vulnerable to HIV (Bowleg et al., 2013a). Masculinity is a social construct determined by how men’s behaviours and actions are judged according to cultural expectations (Fleming et al., 2016; Jacques-Aviñó et al., 2019). It is a male gender role, described as an achieved status characterized by toughness, aggressiveness, stoicism, and sexuality (Brown, Sorrell, and Raffaelli, 2005). Since it is a socially constructed gender identity, it varies from society to society.

Heterosexual ACB men living with HIV are often negatively constructed in scholarly discourse as active transmitters of HIV (Bowleg et al., 2013b; Jacques-Aviñó et al., 2019; Jewkes et al., 2015). Heterosexual Black men have experienced HIV-related criminalization. Black men living with HIV in Canada have been charged with HIV non-disclosure (Mykhalovskiy & Betteridge, 2012). For example, almost half of the people charged with HIV non-disclosure between 2012 and 2016 for which race is known were Black men (Canadian HIV/AIDS Legal Network, 2019). Although the Supreme Court of Canada does not oblige a person with a low viral load (under 1500 copies) to disclose their HIV status (Canadian HIV/AIDS Legal Network, 2019), the media still perpetuates discourse that stigmatizes ACB men (Mykhalovskiy et al., 2020). HIV criminal non-disclosure cases are over-reported and racialized men living with HIV are portrayed as a threat to public health and to the purity of White settlers (Hastings et al., 2020). Similarly, Mykhalovskiy et al. (2020) critique racial profiling and criminalization of HIV non-disclosure, as well as media portrayal of Black men as threats to public health and moral purity of Canada as a nation. Media accounts of criminal HIV non-disclosure cases are often reductionist and culturally insensitive, and rely on sensational language that focuses on negative stereotypes while emphasizing the offenders’ immigrant background and the threat that people living with HIV pose to the general public (Kilty & Bogosavljevic, 2019).

Discourse on Black masculinity has largely hinged on the notion of hypermasculinity, including having multiple sexual partners and avoiding protective measures (Bowleg, 2004; Bowleg et al., 2013b). Given that socialization and behaviour of young men contribute to the spread of HIV, Barker and Ricardo (2005) link masculinity to issues such as multiple sexual partnerships, cross-generational sex, sexual violence against women, non-use of condoms, and low level of HIV awareness. Hypermasculinity may appear positive and complementary and can be related to Black superhumanization bias (BSB). Superhumanization (Black people’s possession of supernatural mental and physical qualities), although seemingly positive, in fact has negative consequences as it can lead to reduced empathy for Black people in need of help (Waytz et al., 2015). Hegemonic masculinity has been correlated with substance abuse and resistance to condom use (Fleming et al., 2016). Indeed, elements of hegemonic masculinity, such as having multiple sexual partners, consuming alcohol before sex, and having unprotected sex, can negatively affect health (Connell, 2012; Peacock et al., 2006). According to Brown et al. (2005), in some extreme cases, living with HIV itself is a symbol of manliness. It has been argued that those who have experienced social or economic discrimination are more likely to engage in risky sex (Bowleg et al., 2013b). Black men have also been described as problematic in terms of opposing homosexuality (Bowleg, 2004). Furthermore, Black men have been alleged to lack responsibility towards children (Watson, 2010) and to be abusive fathers (Meyer and Struthers, 2012). Men living with HIV have been described in the literature as irresponsible since they allegedly do not care for their partners when the latter develop AIDS (Meyer and Struthers, 2012).

Research conducted in Nova Scotia found that Canadian men of African descent experience a greater impact of prostate cancer than white men due to their reliance on sexual prowess to affirm their masculinity in the face of limited access to employment, education, and socio-economic status (Evans et al., 2005). Despite the problematic stereotypes, emerging literature shows that Black men can engage in health-promoting activities. For example, a Canadian research by Husbands et al. (2017) shows that discussing how sex relates to health is part of the masculine identity of heterosexual Black men. Oliffe et al. (2018) discuss how positive male care patterns and relational styles are masculine strengths that can potentially be used to promote positive health outcomes. Hammer and Good (2010) have also associated masculinity with positive attributes, such as physical strength and fitness, although only 9% of participants were Black and the study was United States-based as most research on the subject is.

Therefore, Canadian-based research is needed to explore the resourcefulness of heterosexual ACB masculinity and the degree to which this population adheres to traditional notions of masculinity in ways that positively impact their families and communities. We contend that although some ACB men engage in risky sexual behaviour, homophobia, and other notions of traditional masculinity, ACB men are not incapable of positively influencing health policy. This paper therefore demonstrates how ACB heterosexual men and youth in Ontario perceive masculinity as being responsible in certain aspects of life, including health. The study was conducted to generate knowledge that informs effective community HIV responses which engage heterosexual ACB men and their communities, to examine the social and behavioural vulnerabilities to HIV among heterosexual ACB men, and to identify the individual and structural factors that promote resilience and reduce HIV-related vulnerabilities among ACB men. Using qualitative methods, we explored HIV vulnerability and resilience, including how self-identified heterosexual ACB men and youth understand their vulnerability and resilience, especially in relation to HIV, and how they respond to common gendered and racialized interpretations of Black men’s heterosexuality and masculinity.

## Theoretical framework

The study was guided by critical social theory, which is concerned with social justice and empowering people to overcome the constraints of race, gender, or class (Fay, 1987). Critical social theory has enabled us to elucidate how the dominant notions of Black heterosexuality and masculinity do not address stereotypes applied to ACB men’s behaviour. We also analyzed whether ACB men themselves accept or internalize these stereotypes and narratives. Moreover, we analyzed how the intersectionality of the racial identity of ACB men and youth, their heterosexuality, and masculinity impacts their well-being. This paper highlights the lack of resources necessary for Black males to challenge society’s perceptions of them and to address the harmful consequences of these societal expectations and stereotypes in the same way that more privileged heterosexual men can. Also, as masculine archetypes have been conceived in ways that maintain, justify, or rationalize particular social hierarchies (Rankin and Winsa, 2013), we were interested in how heterosexual ACB men and youth routinely adapt, transform, or resist these masculine archetypes. In this article, we challenge the dominant construct of ACB masculinity as being problematic, and we highlight its potential significance in strengthening community response to HIV and health.

## Research methods

The research was conducted as part of weSpeak, a 5-year multi-site project which aimed to enhance ACB men’s appreciation of the conditions for HIV spread, to increase their commitment to HIV prevention, and to consolidate community networks to end HIV and promote health in ACB communities. The project was implemented by members of the research team^1^, who worked with service providers, policy makers, and community members in four cities: Ottawa, Windsor, London, and Toronto. To ensure trustworthiness, the program was designed and implemented by a research team composed of academic researchers of African descent and local advisory committee members, all of whom met regularly to critically review the data collection process. The team engaged in peer debriefing to discuss study themes as they emerged. This paper reports findings from the Ottawa site.

## Research approach

The study employed a community-based participatory research (CBPR) approach. Members of the research team engaged community members/heterosexual ACB men and stakeholders (service providers, community leaders, policy/decision-makers, and advocates) throughout the life of the project.

## Research participants

This paper is based on qualitative data collected through Focus Group Discussions (FGDs) and individual in-depth interviews (IDIs) with men and youth living with HIV (PHAs), HIV-negative men and youth (non-PHAs), and service providers, all of whom are ACB. Eligibility criteria for participation in the FGDs and IDIs included self-identifying as heterosexual ACB male (including transmen who identify as heterosexual), being at least 16 years old, being PHA or non-PHA, being able to communicate in English or French, and residing in Ottawa. Data collection was preceded by a community engagement campaign. We conducted six FGDs with ACB men and service providers and 16 interviews with ACB men.

## Data collection

Data were collected using FGD and in-depth interview guidelines, at a mutually convenient time and place. Participants were informed of the purpose of the study before each FGD or interview. FGDs explored heterosexual ACB men’s perspectives on their HIV vulnerabilities, as well as strategies to engage our target population in community HIV responses. The in-depth interviews drew from insights and critical issues that emerged from FGDs, and enabled participants to discuss issues related to vulnerability, relationships, sex, and other sensitive or personal issues that could not be effectively captured from FGDs. The interviews and FGDs also assessed how vulnerability, resilience, heterosexuality, and masculinity emerge in everyday experiences. All responses were audio-recorded, and hand-written field notes were taken during data collection to capture any non-verbal cues.

## Data analysis

Our data analysis was guided by critical social theory (Fay, 1987). All FGDs and IDIs audio files were transcribed verbatim, and the typed versions were edited for analytical clarity. We used NVivo software for data management and analysis, and conducted thematic analysis guided by the six-step process of Braun and Clarke (2006). The analysis included development of a coding framework informed by questions from the interview guides and a systematic approach that involved (1) familiarization with the data; (2) generating initial codes; (3) developing a coding tree to guide the coding of transcripts; (4) identifying themes; (5) reviewing, defining, and naming themes; (6) interpreting the narratives and stories; and (7) producing the report—a concise, coherent, logical, and non-repetitive account supported by vivid examples (Boyatzis, 1998). These iterative processes, which are typical of qualitative analysis, ensure that to gain new insights, preliminary themes and interpretations are tested and revised upon further data collection. During data analysis, preconceptions and assumptions were challenged, and a consensus was reached in terms of data interpretation. Thematic analysis was considered an appropriate modality for this study as it is participatory and accessible, and enhances collaborative data analysis and interpretation (Braun and Clarke, 2006).

## Ethical considerations

Prior to the commencement of the study, we obtained ethical clearance from Ottawa Public Health and the University of Ottawa Research Ethics Board (REB). During data collection and analysis, we obtained informed written consent and assured participants of confidentiality. All typed records were kept on a password-protected computer and backup drive. Participants were informed of their right to withdraw their consent at any time. Participants were also provided with the contact details of the University of Ottawa and Ottawa Public Health Ethics Committee for onward reporting of any possible ethical concerns and complaints.

## Results

### Masculinity and family responsibility

The research findings show that heterosexual ACB men and youth associate masculinity with strength. They display their masculinity through their demeanor, the way they dress, and even the music they listen to. For example, during an FGD, one ACB man noted that:

“A man should be strong, never crying, and he should not show any of his feminine side, this is like showing weaknesses. You must always show that you are stronger even though there is someone else stronger than you, who can beat the hell out of you and break all your teeth. So, where is our toughness then? This kind of view holds for now ….” [25+, Adult Non-PHA 027, Ottawa].

However, contrary to previous studies, strength does not equate to violence. Although some ACB men may express their masculinity through aggression and lack of respect towards women, our study participants described men as people who use their strength to defend and protect their family members, including the women in their lives (FGD 02, 25+ Adult Non-PHA). Hence, although previous studies described ACB men as lacking responsibility to take care of their families, this study found that heterosexual ACB men in Ontario viewed their responsibilities towards family and society as an integral part of their masculinity. Participants also noted that being a man is not a matter of age, but of acting with wisdom, including being able to protect family members. One ACB man noted:

“My personal expectations without anything else home…was the way I am built is just to defend. If you speak to my wife wrong, I will show up and address that situation. I don’t care who you are, you can even be bigger than me, but I would show up and address it. If I hear a cry of my child, this starts from a very young age. When I am sleeping and the child is crying, this would cut through my sleep and I would wake up. I am a very heavy sleeper…that is from my very young age. So, my personal expectation is to defend everyone in my …” [FGD 02, 25+ Adult Non-PHA, Ottawa].

The above excerpt shows that strength is both an attribute of masculinity and an important resource for protecting one’s family, including self, wife, and children. During the FGDs, participants noted that although some ACB men may tend to be aggressive and irresponsible, these behaviours cannot be generalized to all Black men. One man noted:

“Being a man means knowing yourself and knowing you have responsibility to your children, to your family, and you have a part to play in the society, and you have to find your rightful part to play in the society. They are men out there who know what it means, and people who are acting foolishly. So, it’s not about your age, it’s about understanding. …” [FGD 02, 25+, Adult Non-PHA, Ottawa].

According to this participant, masculinity is defined more by how a man fulfills his responsibilities than his chronological age. Additionally, some participants suggested that the ‘sexual maturity’ of ACB men is evidenced by those who live up to family and communal expectations to get married, have children, and raise a family. Having a family and taking care of it also enhances ACB men’s perception of their own masculinity.

“When you really become a sexually mature adult, there is that feeling that especially the family wants to see you responsible. It is expected of a man in this age ... to have a wife and children. Children are wealth. The family expects to see first, that you can get married and have children and also take care of them, so there is that pressure there. You need to show that you can have your own family and be in charge” [25+, Adult PHA 035, Ottawa].

This sentiment suggests that heterosexual ACB men living with HIV view masculinity not only in terms of having a wife and children but also in terms of loving and taking care of them. Black masculinity is therefore measured by the ability to be responsible for one’s family.

The findings further show that some ACB men in Ontario refuse to succumb to cultural pressures which compromise family well-being. For example, some participants contributed to household chores, which is atypical for a male in Black households. As one man asserted:

“… it does not matter where you come from, even if you come from Africa, you have to go to the kitchen and help your wife; it doesn’t make you less of a man. You are just a man and you are trying to help out with kids, watching them, cleaning them and doing all these things because it is overwhelming and we don’t have enough time, work and family and all these responsibilities and being an immigrant if you really wanna keep your marriage, you gonna have to participate. You gonna have to work together. That’s the situation in Canada. To me, I don’t take that as you are being weak or you are not showing the strength of being a heterosexual man, no I don’t think so, it is just the nature of life ...” [FGD 06, 25+ Adult PHA, Ottawa].

This participant softens the hegemonic view of the patriarchal and powerful Black man who acts as sole breadwinner and leader of his family. He draws attention instead to a more cooperative Black man who is aware of modern realities, helps in the kitchen, and takes care of children, without perceiving such duties as reducing his masculinity. The participant also remarked that men who contribute to household chores are more likely to have successful marriages, which is another positive departure from the hegemonic patriarchal views on the roles of husband and wife in the household.

We further found that some heterosexual ACB men think about masculinity in terms of their resourcefulness to the community and the nation as a whole. One participant (quoted below) attached his masculinity to ensuring the health, food, and environmental safety for his children.

“I think that my role is being a responsible citizen, a provider for my family, that’s kind of what I look at as my first priority. My other priority is like thinking about the community as a whole. What is my kids gonna do tomorrow? It’s about safe community, safe environment in terms of safety goals and in terms of health, food security? I feel like it is my role and responsibility to make sure that not only me as a person who need to bring food to the table, but also think of diverse ways of how I can help other community and society members, you know to have a living in a safe health environment. That’s kind of how I look at it ...” [25+, Adult Non-PHA, 023].

Overall, ACB men in this study viewed their masculinity positively in relation to their responsibility towards their families and, by extension, towards their communities and nation.

### Economic responsibility

The study found that a man’s ACB or HIV status does not hinder his ability to financially support his family. Heterosexual ACB men define a responsible man as one who affords his family basic living necessities, such as food and housing, in addition to exuding toughness and capability constantly. When asked what it means to be a man, the responses highlighted financial stability, economic responsibility, and working hard.

“… it is not prestige; it is about responsibility.” [25+, Adult PHA 035, Ottawa].

“They like to tell us being a Black man in this world you do extra. If a White man get an A, you have to try to get an A+ … you got to get almost like three times better to get recognized.” [FGD Youth PHA 16-24, Ottawa].

“I think about how I make money. My economic situation has to be so [… hesitation] like it’s a top priority in my life. There are other priorities, but economics is the top priority I cannot lack. I cannot! So, to a heterosexual Black man that I am, it’s very key for me it is to be economically stable, not just for my family but also for my friends, like I said, my friend struggles I want to step in and teach them how to do it right.” [FGD 02, 25+ Adult Non-PHA, Ottawa].

“As for the school I went to, there were probably five Black people in the whole school. I always felt like I have to set the tone instead of the status quo and let them know whatever stereotype they had, it wasn’t true. Because they assumed that I wasn’t smart as the other kids were. So, I always strived to get the highest grades to be the leader in the class.” [FGD Youth PHA 16–24, Ottawa].

“Now, with economics, let’s face it. Everything costs money. So, I need to be…my…I don’t even need to think about the option of not having money. It is ruled out. It is like when I got married, I don’t think about divorce. I don’t allow that option to come…” [FGD 02, 25+ Adult Non-PHA, Ottawa].

“As a man society expect to be providers and protectors. To be a man, that’s what it means: to be a provider and a protector, and you need to lead.” [FGD 02, 25+, Adult Non-PHA, Ottawa].

Although some studies found that ACB men do not provide for their HIV-positive partners (e.g., Meyer and Struthers, 2012), the above interview excerpts illustrate the resourcefulness of ACB men who participated in this study, and show that some ACB men perceive their Black masculinity as an obligation to be financially responsible within and beyond their families. Single ACB young men stressed the importance of working hard as a way of challenging certain stereotypes of ACB people. A participant viewed masculinity as extending beyond physical appearance to include one’s actions and behaviour.

“Basically, it comes down to how do you define yourself as a man, right? So man being man, it’s more than having a penis. It comes down to being able to do things, being able to do physical work, mental work, being able to provide for your family, being able to bring food to the table, being able to keep a roof over your head, living an independent honest life [silence …]. So, to me that’s how I define being manly and again coming down to … typically, Africans are known to be strong, you wanna be strong, right in terms of being strong like the way you walk, the way you look.” [25+, Adult Non-PHA 023, Ottawa].

The above response strongly links masculinity to the ability not only to provide but also to do physical and mental work, and to be an honest individual. This shows that some ACB men have a positive physical and mental perception of masculinity, which incorporates positive ethical attributes such as honesty.

Some participants commented that being Black demands working hard and assuming more responsibility.

“Being a Black [man] is a big responsibility, because everywhere you go, you will be treated differently” [FGD Youth PHA 16–24, Ottawa].

“I never forget one of my [Black] friends, he came to my school at grade nine ... He wanted to do well in everything and having someone like that around, … it helped me confirm that yes, I am on a good path and if I keep on going, I will end up in good place. … It motivated me to become a better person...” [FGD Youth PHA 16–24, Ottawa].

Overall, most participants underscored their want and need to be economically independent and to provide for their families. They perceived a need to do more to prove oneself, on the basis that society has different expectations for heterosexual Black men compared to their non-Black peers.

### Defining masculinity through sexual power

The study also investigated participants’ views on sexual behaviour in view of the prevailing problematic narrative surrounding ACB men. The findings revealed that most participants agreed that while sexual power defines masculinity, such power should be controlled (as captured in the following FGD excerpts):

“Yes, sexual power, being able to abstain, the power to say, okay I may be mature, but I can transfer my testosterone on something else, it can be a good conversation, it can be a good supper, it can be a good talk. …” [25+, Adult Non-PHA 027, Ottawa].

“… a man has to be able to control his instincts and take a step back from a situation and even when he has some sudden sexual needs. We must be able to control that power of ours... I am aware that it takes a lot of work, and sacrifices ...” [25+, Adult Non-PHA 027, Ottawa].

Also, contrary to previous ACB masculinity studies, our study found that heterosexual ACB men in Ontario do consider having protected sex.

“Manhood for me goes with some accountability. Today, it goes with a certain degree of risk. It is not because I have the opportunity that I should jump in without care, without protection without taking into account everything that is done to avoid problems” [25+, Adult Non-PHA 027, Ottawa].

Overall, Black youth and men in this study managed their risk of STI/HIV transmission through a deep commitment to a positive form of masculinity which incorporates a greater attention to self-care, including exercise, and an increased involvement in household chores, including childcare.

## Discussion

Previous studies on Black masculinity have portrayed ACB men as active transmitters of HIV in society (Bowleg et al., 2013b; Jacques-Aviñó et al., 2019; Jewkes et al., 2015). Consequently, Black men have had to find ways to deal with their vulnerabilities in order to improve their health (Gibbs et al., 2015). In the face of limited access to health information and care, some of the coping mechanisms employed by heterosexual ACB men further render them vulnerable. Studies have also characterized Black men with the tendency to have multiple sexual partners and the unwillingness to use protective measures (Bowleg, 2004; Bowleg et al., 2013b). Black men’s adherence to traditional views of masculinity has been associated with negative health outcomes, including increased risk of contracting HIV and AIDS (Connell, 2012; Peacock et al., 2006), as well as negative family interactions, such as becoming an absent or abusive father or husband, due to cultural norms which discourage male involvement in house chores and childcare (Meyer & Struthers, 2012; Watson, 2010). These individual issues and behaviours of ACB men must be analyzed and addressed within the larger context of the structural barriers faced by ACB people, including systemic racism. Thus, efforts to address these issues must include practice and policy interventions that target multiple levels and stakeholders.

In this study, most participants defined masculinity by their ability to provide for, protect, and lead their families. The men underscored being a good provider for their families and being a defender of their wives and children as key characteristics of masculinity. They asserted that ACB men and youth are expected to always be strong, bold, and responsible members of society. The results show that heterosexual ACB masculinity is characterized by having the required strength and ability to protect one’s family members, to raise a family, and to serve as a valuable member of the community, specifically by ensuring access to health and food, environmental safety, and financial stability.

We observed that the view of some participants aligned with patriarchal forms of masculinity and the notion of heterosexual Black men as the breadwinners and heads of their households, which might raise some legitimate concerns about gender inequality. However, their role as providers and protectors is emphasized within the context of responsibility, which is less control-driven and more about ensuring the family’s sustenance and well-being. This is what [FGD 06, 25+ Adult PHA, Ottawa] appears to stress when he underscores that to keep his family unit intact, a man must involve himself in non-traditional household activities (do dishes, clean the house, and take care of children). Perhaps this suggests that for some heterosexual Black men such as this participant, masculinity constitutes role flexibility within his household to accommodate family’s needs, as opposed to being a breadwinner who leaves household duties to his wife and children. These findings challenge scholarly discourse and mainstream notions of Black masculinity as uncontrolled, toxic, risky, or even predatory. Moreover, this positive view of masculinity can be beneficial to ACB men’s well-being and may become a resource for strengthening community responses to HIV and health.

The power aspect of masculinity, as found by our study, has also been identified in previous studies by Brown et al. (2005) and Fields et al. (2015) who associated masculinity with physical strength. Similarly, heterosexual ACB men and youth in our study equated masculinity with physical strength, which they perceived to have positive significance. They acknowledged that while this physical strength is an essential part of manhood, it must be kept in check and appropriately controlled and channeled as a resource for the benefit of their families. This view contradicts previous studies which associated ACB men’s strength with hypersexuality and related vulnerability to HIV infection and spread.

Participants of this study voiced their ability to act and behave responsibly, for instance through participation in household chores or physical exercise. Participants’ understanding and non-subscription to societal stereotypes of hegemonic masculinity adds new insight to the scholarly literature on masculinity among ACB men and youth. The findings demonstrate that ACB men do not accept or internalize such stereotypes. On the contrary, they are aware of the narrative that hypersexualizes them, and this research program gave them an opportunity to verbally contest these masculine archetypes.

Furthermore, the participants’ perception of masculinity as including the ability to have a family and protect and defend their children is a new and powerful insight. In contrast to previous studies by Watson (2010) and Meyer and Struthers (2012), our study demonstrates that ACB men do care for their children and partners. The findings therefore contradict previous generalizations about heterosexual ACB men and suggest that some heterosexual ACB men use their strength for the benefit of their families and society at large. Such positive men can be empowered to influence the necessary changes among fellow ACB men and youth. This finding implies that heterosexual ACB men’s masculinity can be mobilized as a resource to achieve the desired health implications. In addition to having responsibilities toward the family and society, being a man also means being a leader, provider, and a protector. Earning is therefore among the priorities of ACB men, since income enables them to care for their families.

Although masculinity has been linked to higher health risks (Bowleg et al., 2013a; Jacques-Aviñó et al., 2019), including avoidance to seek healthcare due to anti-Black bias in medical establishments (Randall, 1995), our research found that heterosexual ACB men are concerned about their health. However, ACB men still need resources to motivate them to meaningfully engage in health and HIV programs. The findings of this study, which sought and documented the perceptions of heterosexual ACB men on masculinity, demonstrate that ACB men’s problematic framing of issues facing them is possibly attributed to the silencing of their voices. This research has given heterosexual ACB men and youth a platform to voice their perspectives on masculinity, which is a powerful resource for counteracting the negative constructions often found in masculinity discourse about racialized men.

It is important to note that throughout our interviews and FGDs, participants were not prompted to speak about their experiences of living with HIV because we expected them to volunteer this information without being asked. Unfortunately, for the Ottawa site, we were unable to capture data with participants’ views about how living with HIV impacted on their representations of masculinity. This is something that can be addressed in future studies on Black masculinity.

## Conclusion

Dominant discourse (particularly in the media) seems to project ACB men in a negative light, for example, highlighting risky sexual behaviour and characterizing them as irresponsible, while neglecting the positive aspects of their everyday behaviour. The literature tends to paint the picture of ACB masculinity in terms of sexual prowess, physical strength, and engagement in risky sexual behaviour. Our research findings refute such stereotypes and generalizations. While negative and risky behaviour by any man is an important health issue that demands attention, ACB men and youth in our study do not conform to such problematic framing, given their clearly stated perception that masculinity embodies not only strength and power but also the ability to control and channel them appropriately. ACB youth and men are capable of avoiding risky sexual behaviour and engaging in healthy alternatives, including physical exercise and helping with household chores. These findings refute the stereotype of ACB men as highly vulnerable to HIV, and by implication, as key transmitters of HIV through their uncontrollable sexual behaviour. Heterosexual ACB men view masculinity as being responsible, working hard, having economic stability to provide for their families, living an independent and honest life, and being a valuable member of the community and society at large. This new understanding of masculinity among heterosexual ACB men can be useful toward theorizing more complex and nuanced understandings of masculinity in general.

## Contributions to knowledge

What does this study add to existing knowledge?

- To the best of our knowledge, this is the first study that challenges the negative discourse that surrounds heterosexual Black masculinity in relation to HIV vulnerability.
- The paper highlights how heterosexual Black male participants’ self-affirming narratives resist common gendered and sexually racialized interpretations of Black men’s heterosexuality and masculinity.

What are the key implications for public health interventions, practice or policy?

- There is an evidence gap on how Black heterosexual masculinity is understood in the context of HIV vulnerability. Hence, we recommend a national study on this topic since individual ACB males’ issues must be analyzed and addressed within the larger context of the structural barriers, including systemic racism.
- We also recommend a holistic approach to innovative policy and practice targeting stakeholders from various sectors at different levels.
- Additionally, it is important to acknowledge and support community-level initiatives to enhance the self-affirming narratives of heterosexual ACB males that resist problematic gendered and sexually racialized interpretations of Black men’s heterosexuality and masculinity.

## Data Availability

Supplementary material is available from the corresponding author upon request.

## Footnotes

1 Josephine P. Wong (nominated principal investigator), Winston Husband (principal investigator), Josephine Etowa (principal investigator), Isaac Luginaah (principal investigator), Francisca Omorodion (principal investigator), Alexander Asamoah, Kenneth Fung, Carl James, Jelani Kerr, Erica Lawson, Hugues Loemba, Henry Luyombya, Lance McCready, Frank McGee, Paul Mkandawire, Wesley Oakes, Fanta Ongoiba, Valérie Pierre-Pierre, Maurice Poon, Wangari Tharao, Zhaida Uddin, Mandana Vahabi, Charmaine Williams. Collaborators: Racquel Bremmer, Garfield Durrant, Ahmed Bechir Habré, Francisco Ibanez-Carrasco, Brian Lester, Barbara MacPherson, Khaled Salam

## Code availability

Code details are available from the corresponding author upon request.
